# Assessment of Postural Stability in Semi-Open Prisoners: A Pilot Study

**DOI:** 10.3390/jcm14186399

**Published:** 2025-09-10

**Authors:** Michalina Błażkiewicz, Jacek Wąsik, Justyna Kędziorek, Wiktoria Bandura, Jakub Kacprzak, Kamil Radecki, Karolina Kowalewska, Dariusz Mosler

**Affiliations:** 1 Faculty of Rehabilitation, The Józef Piłsudski University of Physical Education in Warsaw, 00-968 Warsaw, Poland; justyna.kedziorek@awf.edu.pl (J.K.); wiktoria.bandura@awf.edu.pl (W.B.); 2 Institute of Physical Culture Sciences, Jan Długosz University in Częstochowa, 42-200 Częstochowa, Poland; jakub.kacprzak@doktorant.ujd.edu.pl (J.K.); kamil_radecki@onet.pl (K.R.); kowalewskamaja@wp.pl (K.K.); d.mosler@ujd.edu.pl (D.M.)

**Keywords:** postural control, linear measures, nonlinear measures, body balance, substance dependence

## Abstract

**Background/Objectives**: This study investigated postural stability in male inmates of a semi-open correctional facility, with a specific focus on comparing individuals with and without a history of substance dependence. The aim was to identify how addiction-related neurophysiological changes impact postural control under varying sensory and biomechanical demands. **Methods**: A total of 47 adult male prisoners (mean age: 24.3 years) participated in this study. Nineteen inmates had a documented history of alcohol or drug dependence (addicted group), while twenty-eight had no such history (non-addicted group). All participants were physically able and free of neurological disorders. Postural control was assessed using a stabilometric platform and wireless IMU across six 30 s standing tasks of varying difficulty (bipedal/unipedal stance and eyes open/closed). Linear (center of pressure path and ellipse area) and nonlinear (sample entropy, fractal dimension, and the Lyapunov exponent) sway metrics were analyzed, along with trunk kinematics from IMU data. This study received institutional ethical approval; trial registration was not required. **Results**: The addicted group showed greater instability, especially in the eyes-closed and single-leg tasks, with increased sway and irregularity in the anterior–posterior direction. IMU data indicated altered trunk motion, suggesting impaired neuromuscular control. In contrast, non-addicted individuals demonstrated more efficient, targeted postural strategies, while addicted participants relied on broader, less selective movements, possibly reflecting compensatory or neuroadaptive changes from substance use. **Conclusions**: Substance dependence is associated with compromised postural stability in incarcerated men. Balance assessments may be valuable for detecting functional impairments and guiding rehabilitation within prison healthcare systems.

## 1. Introduction

Postural control is a fundamental aspect of human motor function, essential for maintaining balance and orientation during both static and dynamic activities [1,2,3,4,5]. It involves the complex integration of sensory information, primarily from the visual, vestibular, and proprioceptive systems, along with motor responses and higher-order cognitive processes within the central nervous system [6]. Any disruption to these systems, whether from neurological damage, chronic illness, or behavioral factors, can result in impaired balance, increased fall risk, and reduced functional independence [7].

Among behavioral factors, substance dependence is a well-established contributor to compromised postural stability. Chronic consumption of alcohol and other psychoactive substances has been shown to adversely affect neurophysiological functioning [8]. For instance, alcohol has neurotoxic effects on the cerebellum, critical for coordinating movement and balance, leading to persistent postural instability that can continue even after cessation of use [9,10]. Similarly, prolonged use of central nervous system depressants or stimulants, such as opioids, benzodiazepines, and amphetamines, can impair neuromuscular coordination, sensory integration, and executive function [11,12]. These effects can be subtle but detectable, especially under difficult balance conditions such as standing with eyes closed or performing one-legged positions [6].

In correctional settings, substance use disorders (SUDs) are highly prevalent but often under diagnosed and undertreated. Studies indicate that a majority of incarcerated individuals have a history of substance use, with many meeting diagnostic criteria for SUD. However, the associated functional impairments—including motor and postural deficits—receive limited attention in prison healthcare systems [13]. Current rehabilitation strategies in these environments largely focus on psychological and behavioral interventions, frequently neglecting the physical and neurological consequences of long-term substance abuse [6].

Inmates housed in semi-open penitentiaries represent a particularly relevant subgroup. These facilities permit greater autonomy, mobility, and participation in vocational or rehabilitative activities, offering a more ecologically valid setting for assessing functional abilities than high-security institutions [14]. The increased physical demands and environmental variability in semi-open settings may place a greater burden on postural control systems, making balance assessments especially informative in this context [15]. Furthermore, prisoners in such facilities are often closer to reintegration into society, and postural deficits may limit their capacity to engage in daily, vocational, or training activities, making functional assessment critical for rehabilitation planning.

This population is of particular interest because it combines several risk factors for impaired balance: substance use history, confinement-related inactivity, limited access to medical care, and environmental unpredictability. Postural assessment in incarcerated individuals can serve not only as a functional health indicator but also as a screening tool for fall risk or neuromuscular dysfunction [16,17].

Clinically, impaired postural control in this population may increase the risk of falls, injury, and functional decline, potentially limiting participation in rehabilitation programs and daily activities. This, in turn, may affect readiness for reintegration into society and overall long-term health outcomes. Identifying and addressing such deficits early through tailored interventions could improve both safety and rehabilitation success. These observations align with the emerging literature showing that incarcerated individuals often demonstrate lower physical function, higher frailty risk, and sensorimotor alterations than the general population, even at younger ages [18,19]. Functional testing, including postural assessment, has been recommended as a component of prison healthcare to identify undetected impairments that can influence long-term outcomes and readiness for rehabilitation.

Evaluating postural stability in this population can yield valuable insights into physical readiness for reintegration, injury risk, and the need for targeted interventions. Additionally, recent studies emphasize the effectiveness of compensatory exercises in correcting postural deficits among young athletes, an approach that may also benefit incarcerated individuals experiencing substance-related motor impairments [20,21].

Despite the central role of postural control in both health and rehabilitation outcomes, few studies have investigated balance performance in incarcerated populations, particularly concerning substance use history [22]. Even fewer [23,24] have employed objective, standardized measurement tools such as stabilometric platforms, which track displacement of the center of pressure (CoP) under the feet. These platforms enable detailed analysis of body sway in the anterior–posterior (AP) and mediolateral (ML) directions, providing reliable indicators of postural control mechanisms [25].

To quantify postural sway, commonly used linear measures include CoP velocity, CoP path length, and the ellipse sway area. While these metrics are informative, they may not fully capture the nonlinear dynamics and complexity inherent in the postural control system [26]. Therefore, nonlinear measures are increasingly employed to complement traditional approaches.

Nonlinear features such as sample entropy (SampEn), fractal dimension (FD), and the Lyapunov exponent (LyE) offer insights into different components of neuromotor control that are not evident in linear metrics [26]. SampEn captures the predictability and complexity of CoP fluctuations. In the context of neuromotor control, lower entropy suggests a more rigid, less adaptive control system, often observed in pathological states, while higher entropy indicates greater adaptability and responsiveness to internal or external perturbations [25,27]. FD reflects the geometrical complexity of CoP trajectories over time. A more complex trajectory (i.e., higher FD) generally corresponds to a more adaptable postural control strategy, able to engage multiple motor degrees of freedom to maintain balance. Reduced FD values are linked with simplified, potentially inefficient postural strategies, often found in older adults or those with neurological impairments [5,25]. LyE quantifies the sensitivity of the system to small disturbances, which is central to assessing dynamic stability. A higher LyE suggests that the postural system is more capable of adapting to perturbations and maintaining balance, a marker of robust control. In contrast, a lower LyE value implies decreased responsiveness and adaptability, potentially reflecting neuromuscular degradation due to substance exposure [25,28]. These nonlinear dynamics are particularly suited to detecting postural changes resulting from a history of addiction, as substance abuse often leads to long-lasting alterations in sensorimotor integration and cortical–subcortical pathways involved in balance regulation [8,29]. The nuanced impairments caused by psychoactive substances may not significantly affect average sway magnitude but can disrupt the underlying temporal structure of balance control, precisely what nonlinear measures are designed to detect.

Previous studies have validated the use of these nonlinear metrics in various clinical populations, including individuals with Parkinson’s disease, multiple sclerosis, stroke, and frailty, where they have revealed subtle motor instabilities undetected by linear metrics [30,31,32,33]. These findings underscore the clinical relevance of nonlinear analyses, as they are capable of capturing diminished adaptability, reduced complexity, and impaired dynamic stability that traditional measures may overlook. In populations with alcohol dependence, nonlinear methods have been shown to predict relapse by modeling the dynamics of behavior change as a complex, self-organizing system sensitive to initial conditions and perturbation [34]. This suggests that nonlinear measures may serve as early indicators of neuromotor dysfunction linked to chronic substance use, offering valuable prognostic and diagnostic utility in both clinical and correctional health settings.

As Kędziorek and Błażkiewicz [25] emphasize, the inclusion of nonlinear metrics is particularly valuable in detecting temporal patterns and subtle instabilities in CoP signals, especially across AP and ML axes. These measures provide a deeper understanding of the regularity, adaptability, and robustness of the postural control system. In summary, integrating nonlinear features allows for a more sensitive, mechanistically informed assessment of postural stability, capable of detecting the persistent effects of neurotoxic substances on motor control.

Addressing this research gap is essential for informing evidence-based rehabilitation strategies that target both the cognitive–behavioral and physical consequences of substance dependence. Doing so could improve the overall health and reintegration prospects of incarcerated individuals and enhance the efficacy of correctional healthcare systems.

Therefore, the primary objective of this study was to assess postural control among male inmates housed in a semi-open correctional facility, with a specific focus on comparing individuals with and without a history of alcohol or drug dependence. Moreover, it was hypothesized that individuals with a history of substance dependence would demonstrate significantly impaired postural control compared to non-addicted inmates, particularly under conditions that challenge sensory and biomechanical systems.

## 2. Materials and Methods

### 2.1. Study Participants: Key Characteristics

This study was an observational cross-sectional comparative study. The research was conducted over two distinct sessions, held on 21 and 28 March 2025, with a sample of 47 male inmates from a semi-open prison (Table 1). Participants were divided into groups based on addiction status: 19 individuals with a documented history of addiction and 28 without. The addicted group consisted of individuals who were either awaiting, undergoing, or had completed treatment for alcohol, psychoactive substance, or other addictions. Therefore, in this paper, the term refers to people who may exhibit alcohol or substance use disorders.

The inclusion criteria for this study comprised male inmates housed in a semi-open facility who were over 18 years of age, physically capable of performing standing tasks, and without diagnosed neurological disorders. Individuals were excluded if they refused to participate or had contraindications for the standing balance assessment. Additionally, those with documented neurological disorders such as epilepsy or Parkinson’s disease, as well as individuals experiencing acute psychiatric episodes at the time of testing, were excluded from this study. None of the participants were engaged in formal balance or motor rehabilitation programs at the time of testing.

The Shapiro–Wilk test indicated non-normal distributions for the parameters in Table 1; therefore, the Mann–Whitney U test was used and showed no significant differences between groups (*p* > 0.05).

Among all participants, 34% had completed secondary education, 34% had received vocational training, 27% had primary education, and only 4% held a higher education degree. Regarding marital status, 57% were single, 14% were married, 21% were divorced, and 6% were in a domestic partnership.

Within the group of addicted prisoners, 26% had completed secondary education, 36% had vocational training, 31% had primary education, and 5% held a higher education degree. The marital status of this group showed that 42% were single, 5% were married, 42% were divorced, and 10% were in a domestic partnership. Among non-addicted prisoners, 39% had secondary education, 32% vocational training, 25% primary education, and only 3% higher education. Regarding marital status, 67% were single, 21% were married, 7% were divorced, and 3% were in a domestic partnership.

Definition of addiction history: Participants were classified based on prison medical records, confirming diagnosis or documented history of substance use disorder (alcohol or psychoactive substances). This information was not solely self-reported.

This study was approved by the University Institutional Review Board (Reference No. SKE01-15/2023) and conducted following the ethical guidelines of the Declaration of Helsinki. All participants were fully informed about this study’s objectives and procedures before participation.

#### Characteristics of the Addicted Inmates

Of the 19 inmates classified in the addiction group, 12 were addicted to alcohol, 6 to psychoactive substances other than alcohol, and 1 to both alcohol and drugs. The duration of substance abuse ranged from 2 to nearly 30 years. One individual is currently undergoing therapy, thirteen have completed therapy, one is awaiting admission, and three are abstinent without the need for further treatment (Table 2).

Most alcohol-dependent inmates reported abuse lasting between 3 and 30 years. One individual is still undergoing therapy at the Therapeutic Unit of the Penitentiary Facility (OT Wojkowice), and six others have completed therapy at the same facility, with engagement levels ranging from moderate to high. Two individuals received therapy at other centers (OT Jasło). Three inmates were not referred for therapy: one due to long-term abstinence (confirmed at 6 years), another due to previous outpatient treatment, and one due to very poor therapy outcomes. Several alcohol-addicted individuals participate in Alcoholics Anonymous groups and re-adaptation programs like “*Alcohol—Enemy of Roads.*”

Among those addicted to psychoactive substances, the average duration of use ranged from 2 to 5 years. Substances included α-PVP, amphetamines, and cannabinoids. Three individuals completed therapy in specialized units with moderate to high engagement. One inmate is awaiting court-mandated treatment, and two others are in confirmed abstinence: one after 1.5 years drug-free and participation in a preventive program, and another whose three-year abuse period ended before incarceration.

One inmate diagnosed with both alcohol and drug addiction completed alcohol-focused therapy at the Therapeutic Unit of the Penitentiary Facility with high engagement. He remains active in Alcoholics Anonymous meetings.

### 2.2. Measurement Procedures

Before data collection, all participants were thoroughly informed about the experimental procedures. Additionally, abstinence at the time of testing was confirmed by prison healthcare staff. While some data on the duration of abstinence were available, it was not consistently documented across participants, which constitutes a limitation of this study.

**Figure 1:**
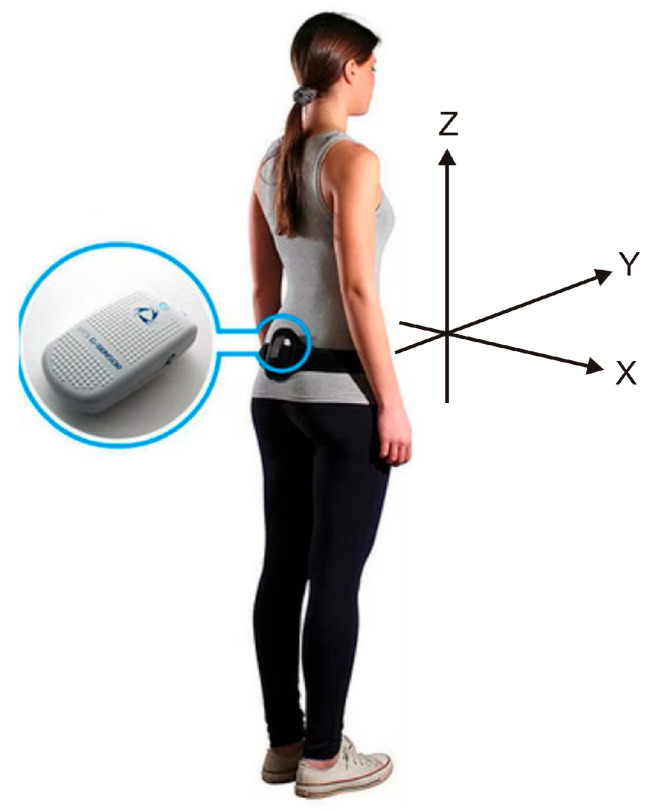
Graphical representation of the BTS G-Sensor (modified from https://www.nept.us/bts-g-walk-p-walk, accessed on 19 June 2025). The X-axis indicates mediolateral (ML) tilt of the trunk, with positive values representing rightward tilt; the Y-axis represents anterior–posterior (AP) movement, with positive values indicating forward motion; and the Z-axis denotes vertical movement, with positive values indicating upward displacement.

Postural control was evaluated using two devices: (1) the STANIAK JB platform (Zbigniew Staniak Jba, Warsaw, Poland), operating at a sampling frequency of 100 Hz, and (2) the BTS G-Sensor (BTS Bioengineering S.p.A., Garbagnate Milanese, Italy), a compact, wireless inertial measurement unit (IMU) designed for kinematic analysis in both clinical and research settings. The system features a lightweight design (37 g) and compact dimensions (70 mm × 40 mm × 18 mm), integrating high-precision inertial sensors: a triaxial accelerometer (16-bit resolution per axis, configurable sensitivity: ±2/±4/±8/±16 g), a triaxial gyroscope (16-bit resolution per axis, adjustable range: ±250/±500/±1000/±2000 deg/s), and a triaxial magnetometer (13-bit resolution, ±1200 µT range). The device was securely fastened to the participant’s waist using a semi-elastic belt (Figure 1), allowing for non-invasive measurement of trunk kinematics. Data were acquired at 100 Hz and transmitted wirelessly via Bluetooth® to a host computer for real-time monitoring and subsequent analysis.

All assessments were conducted between 9:00 a.m. and 12:00 p.m. to control for circadian effects. Each participant performed six 30 s standing trials, with a one-minute rest interval between trials. Trials included both eyes-open and eyes-closed conditions. During each trial, participants wore sports shoes that did not cover their ankles and stood with their arms relaxed at their sides. In the eyes-open trials, participants focused on a fixed point at eye level, approximately 2 m away. The trials were performed in the following order: (1) bipedal standing with eyes open (2eo); (2) bipedal standing with eyes closed (2ec); (3) right-leg standing with eyes open (eoR), (4) left-leg standing with eyes open (eoL), (5) right-leg standing with eyes closed (ecR), and (6) left-leg standing with eyes closed (ecL). Each of the six conditions was performed once, in a fixed sequence.

### 2.3. Linear and Nonlinear Measures

From the BTS G-Sensor, the median values of waveform data (3000 data points) were analyzed for nine parameters. These included angular acceleration in the X (mediolateral), Y (anterior–posterior), and Z (perpendicular) directions, expressed in degrees per second squared [deg/s^2^]. These parameters represent how quickly angular velocity changes over time. Angular velocity itself was also recorded in degrees per second [deg/s] for the X, Y, and Z axes. Finally, orientation angles—roll [deg], pitch [deg], and yaw [deg]—were extracted, representing rotations around the X-, Y-, and Z-axes, respectively.

From the STANIAK JB platform, four linear parameters were extracted for each individual across all six trials. These included the total center of pressure path length (CoP_total), as well as the path lengths in the anterior–posterior (CoP_AP) and mediolateral (CoP_ML) directions, and the surface area of the 95% confidence ellipse (ellipse). Additionally, a 30 s time series of CoP displacements in both the AP and ML directions was exported separately, with each series comprising 3000 data points. Nonlinear measures were then calculated from these time series using MATLAB R2021a (MathWorks, Natick, MA, USA), including sample entropy (SampEn), fractal dimension (FD), and the Lyapunov exponent (LyE).

#### 2.3.1. Sample Entropy (SampEn)

Sample entropy is a metric used to assess the complexity of time series data by estimating the probability that sequences which are similar for a certain length will remain similar when that length is increased by one. It is mathematically defined as $SampEn\left(N,m,r\right)=-ln\left[\frac{B^{m+1}\left(r\right)}{B^{m}\left(r\right)}\right]$, where

$B^{m}\left(r\right)$ denotes the average count of similar pairs of sequences of length mmm that fall within a predefined tolerance *r*.

$B^{m+1}\left(r\right)$ represents the corresponding average for sequences of length *m* + 1.

To ensure consistency across datasets, the tolerance *r* is typically set as 0.2 times the standard deviation of the signal.

In this study, sample entropy was computed using MATLAB R2021a, employing scripts sourced from the PhysioNet library [35]. The default parameters were applied, with *m* = 2 and *r* = 0.2 x (standard deviation of the signal) [36].

The calculation process involved segmenting the time series into overlapping windows of length mmm and then counting how many of these segments were similar to others within the specified tolerance. This yielded the value $B^{m}\left(r\right)$. The same method was repeated for sequences of length *m* + 1, obtaining $B^{m+1}\left(r\right)$. Finally, the sample entropy was derived by computing the negative natural logarithm of the ratio between the two average counts.

This method provides an indication of the signal’s regularity and unpredictability. Lower SampEn values reflect more repetitive or predictable patterns, whereas higher values suggest a greater degree of complexity and randomness.

#### 2.3.2. Fractal Dimension (FD) Estimation Using the Higuchi Algorithm

To determine the fractal dimension (FD) of the time series, the Higuchi method [37] was applied. This approach constructs multiple sub-series from the original signal $X=x\left[1\right],x\left[2\right],\ldots ,x[N]$ by sampling data points at fixed intervals based on a scaling parameter *k.* Each sub-series was defined as

$$X_{k}^{m}=x\left[m\right],x\left[m+k\right],x\left[m+2k\right],\ldots ,x\left[m+int\left(\frac{N-m}{k}\right)\cdot k\right],$$

Here, *m* and *k* are positive integers, with *m* = 1, 2, …, *k*, and $int\left(\frac{N-m}{k}\right)$ represents the integer part of $\left(\frac{N-m}{k}\right)$. The parameter *k* controls the spacing between selected points, effectively setting the time scale of the sub-series.

The length of each sub-series $L\left(m,k\right)$ is computed by summing the absolute differences between successive points and then normalizing the result to account for the scale and the number of segments:

$$L\left(m,k\right)=\left(\sum_{i=1}^{int\left(\frac{N-m}{k}\right)}\left|x\left[m+ik\right]-x\left[m+\left(i-1\right)k\right]\right|\right)\cdot \left(\frac{N-1}{int\left(\frac{N-m}{k}\right)k^{2}}\right),$$

For each value of *k*, the average curve length *L(k)* is obtained by averaging $L\left(m,k\right)$ across all valid *m* values:

$$L\left(k\right)=\frac{1}{k}\sum_{m=1}^{k}L\left(m,k\right).$$

To estimate the fractal dimension, the values of *L*(*k*) are plotted against 1/*k* on a log-log graph. The relationship typically forms a straight line, and the slope of this line corresponds to the fractal dimension of the signal. This slope is estimated using linear regression in the least-squares sense, applied to the points $\left(\ln\left(\frac{1}{k}\right),ln\left(L(k)\right)\right)$.

Selecting an appropriate value for the maximum scale *k_max_* is crucial. To identify this, FD was computed over a range of *k_max_* values. The saturation point, where the FD value stabilized, was used to choose the optimal *k_max_*. In this study, a value of *k_max_* = 100 was selected, following the approach in [38].

#### 2.3.3. The Lyapunov Exponent (LyE)

The Lyapunov exponent (LyE) can be estimated from a one-dimensional time series using a method developed by Wolf et al. [39]. This approach determines the rate at which nearby points diverge from each other in phase space, a key indicator of chaos.

The process begins by selecting a starting point in the time series and finding a close neighbor. The algorithm then monitors how the distance between these two points changes over time. By repeatedly measuring this separation over consecutive steps, the method captures the rate of divergence.

The LyE is derived from this exponential rate of separation. A positive LyE signifies a chaotic system, where even tiny differences in initial conditions lead to exponentially diverging trajectories. Conversely, a negative LyE suggests a stable system, where trajectories converge. By repeating this procedure across the entire time series, the algorithm calculates the dominant LyE, revealing the system’s overall sensitivity to initial conditions and its chaotic characteristics.

### 2.4. Data Analysis

All statistical analyses were performed using Statistica version 12 (StatSoft, Tulsa, OK, USA). A significance level of *p* < 0.05 was used for all tests. The Shapiro–Wilk test was employed to check the normality of the distribution for all parameters, including both linear and nonlinear measures.

First, the Mann–Whitney U test was used to examine whether the addicted and non-addicted groups differed in postural control outcomes, as measured by both linear and nonlinear parameters.

To compute the effect size for a Mann–Whitney U test, the following formula was used: $r=\frac{Z}{\sqrt{N}}$, where Z is the Z-score from the test and N is the total number of observations. In this case, N = 47. The effect size interpretation was as follows: r < 0.3—small effect; 0.3 ≤ r ≤ 0.5—medium effect; and r > 0.5—large effect [40].

In the final stage of analysis, correlations between linear and nonlinear postural parameters and IMU data were computed using MATLAB R2021a. These analyses were conducted separately for individuals with and without addiction, across six balance conditions: 2eo, 2ec, eoR, eoL, ecR, and ecL. Only statistically significant correlations (*p* < 0.05) are reported in this study.

The correlation coefficient reflects both the strength and direction of the relationship between two variables. A positive correlation indicates that as one variable increases, the other tends to increase as well [41]. In contrast, a negative correlation suggests that an increase in one variable is associated with a decrease in the other. The absolute value of the coefficient determines the strength of the relationship.

In general, correlations between 0.10 and 0.30 are considered weak, values from 0.30 to 0.50 indicate a moderate association, and those ranging from 0.50 to 0.70 are regarded as strong. Correlations exceeding 0.70 are considered very strong, while values approaching 1.00 suggest an extremely strong or nearly perfect relationship. Correlation values near zero imply little to no association between variables [41].

## 3. Results

### 3.1. Linear Parameters

Table 3 compares linear postural sway parameters between the addicted and non-addicted groups across six balance conditions (2eo, 2ec, eoR, eoL, ecR, and ecL). Parameters analyzed include the total center of pressure path length (CoP_total), path lengths in the mediolateral (CoP_ML) and anterior–posterior (CoP_AP) directions, and the ellipse surface area representing sway (ellipse). The results show no significant differences during bipedal standing with eyes open (2eo). However, during bipedal standing with eyes closed (2ec), the addicted group exhibited significantly greater CoP_total, CoP_AP, and ellipse area, indicating increased postural sway. Similarly, during single-leg standing with eyes open (eoR and eoL), the addicted group demonstrated significantly larger CoP_total, CoP_ML, and CoP_AP values. The most pronounced differences occurred during single-leg standing with eyes closed (ecR and ecL), where the addicted group showed significantly greater sway across almost all parameters, with moderate to large effect sizes. These results suggest that addiction is associated with impaired postural stability, particularly under more challenging conditions that reduce visual and proprioceptive input, highlighting deficits in neuromuscular control during balance tasks.

### 3.2. Nonlinear Parameters

Table 4 compares nonlinear postural sway parameters between addicted and non-addicted individuals across various balance conditions. Key measures include sample entropy (SampEn), fractal dimension (FD), and the Lyapunov exponent (LyE) in both mediolateral (ML) and anterior–posterior (AP) directions under six postural tasks.

Overall, most comparisons revealed no statistically significant differences between groups, suggesting relatively similar postural control dynamics. However, notable exceptions emerged under more challenging balance conditions. Specifically, during bipedal standing with eyes closed (Trial 2ec), significant differences were observed for SampEn_AP (*p* = 0.04, r = −0.30) and LyE_AP (*p* = 0.01, r = −0.35). In both cases, addicted individuals exhibited higher values, indicating greater irregularity and higher divergence (instability) in sway patterns in the AP direction compared to non-addicted individuals. These findings suggest reduced postural control stability in addicted individuals when visual input is removed.

Other near-significant results, such as for FD_AP in Trial 2ec (*p* = 0.05, r = −0.28) and FD_AP in Trial 2eo (*p* = 0.06, r = −0.27), also hint at increased complexity in sway among addicted individuals, though they did not meet conventional significance thresholds.

No meaningful differences were found in less demanding conditions (e.g., bipedal eyes open) or in tasks with more complex motor demands (e.g., single-leg stance), possibly due to higher variability or compensatory mechanisms across individuals.

In summary, addicted individuals show signs of impaired postural control primarily under sensory-challenged conditions, particularly in the AP direction. These differences may reflect subtle neuromotor deficits associated with addiction, which become evident when proprioceptive and vestibular systems are taxed in the absence of visual input.

### 3.3. Accelerometer Characteristics

An analysis of the IMU data revealed several statistically significant differences between the addicted and non-addicted groups during various balance tasks (Table 5). In the 2eo condition, significant differences were observed in angular acceleration along all three axes. The non-addicted group showed higher X-axis acceleration (Z = 2.00, *p* = 0.04, *r* = 0.29), while the addicted group exhibited greater forward (Y-axis) acceleration in the negative direction (Z = 2.42, *p* = 0.01, *r* = 0.35) and upward (Z-axis) acceleration (Z = −2.49, *p* = 0.01, *r* = −0.36). Additionally, a significant difference in pitch angle was found, with the addicted group displaying a more upright posture (Z = −2.30, *p* = 0.02, *r* = −0.34).

In the eoR condition, a significant increase in gyro X angular velocity was found in the addicted group (Z = −1.95, *p* = 0.04, *r* = −0.28), suggesting greater mediolateral instability.

In the ecL condition, the addicted group demonstrated significantly higher Z-axis angular acceleration (Z = −1.99, *p* = 0.04, *r* = −0.29), indicating increased upward acceleration possibly related to postural adjustments.

No significant differences were found between the groups in the 2ec, eoL, and ecR conditions. Across most variables, effect sizes ranged from small to moderate, suggesting meaningful, although not large, group differences in select stability parameters during tasks with reduced sensory input or increased postural demand.

### 3.4. Correlation Between Linear and Nonlinear Parameters and IMU Data

This section explores the relationships between postural control measures and inertial measurement unit (IMU) data in individuals with and without a history of addiction.

**Figure 2:**
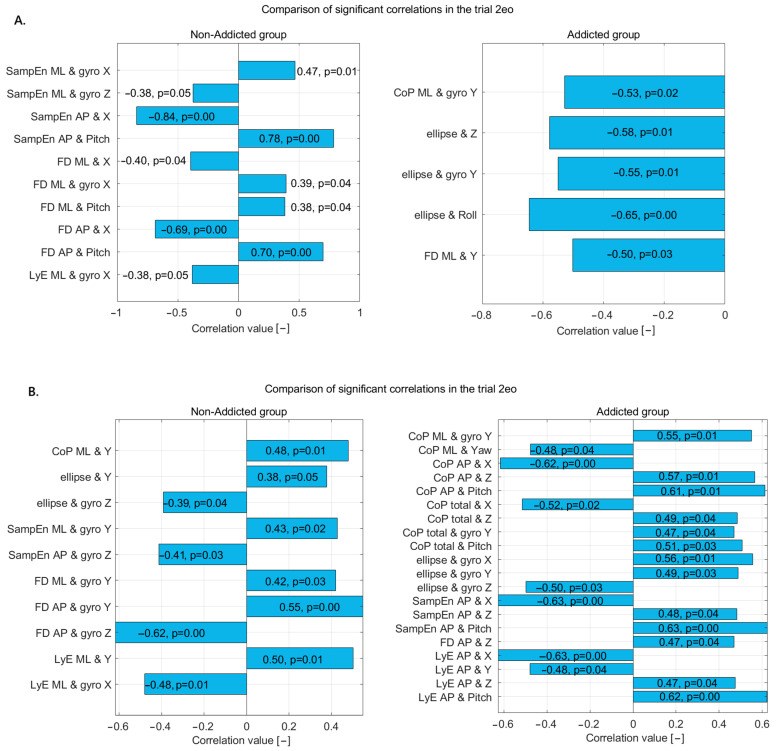
Statistically significant correlations in non-addicted and addicted groups during (**A**) the 2eo trial and (**B**) the 2ec trial—correlation coefficients and *p*-values displayed on bars.

In the 2eo trial (Figure 2A), the non-addicted group exhibited several significant correlations between nonlinear postural measures and motion-related variables. SampEn_ML showed a moderate positive correlation with gyro X, indicating that increased sway complexity in the ML direction is associated with higher rotational movement around the X-axis. Conversely, SampEn_ML had a weaker negative correlation with gyro Z, suggesting that reduced vertical rotation is linked with increased sway complexity. For the AP direction, SampEn demonstrated a very strong negative correlation with linear displacement along the X-axis and a strong positive correlation with pitch, implying that more predictable AP sway is associated with increased forward–backward translation and reduced tilting motion.

FD_ML showed a moderate negative correlation with linear X and moderate positive correlations with gyro X and pitch, suggesting that sway complexity in the ML direction is influenced by both translational and rotational dynamics. FD_AP exhibited a strong negative correlation with linear X and a strong positive correlation with pitch, reinforcing the idea that increased complexity in AP sway is linked to decreased linear displacement but increased rotational tilt. LyE_ML also showed a moderate negative correlation with gyro X, suggesting that lower divergence in ML sway is associated with higher rotational motion around the X-axis.

In the addicted group, a different set of patterns emerged. CoP_ML had a moderate negative correlation with gyro Y, indicating that reduced rotation around the Y-axis is associated with increased ML sway. The ellipse area, representing overall sway spread, showed moderate to strong negative correlations with Z displacement, gyro Y, and roll, implying that greater sway extent is associated with reduced vertical movement, lateral rotation, and side-to-side tilting. Additionally, FD_ML showed a moderate negative correlation with linear Y, suggesting that increased sway complexity in the ML direction is related to reduced vertical displacement.

These results highlight distinct postural control characteristics between the two groups. The non-addicted individuals showed strong associations between entropy and complexity measures with linear X movement and pitch, reflecting a coordinated use of translational and tilting motions for postural stability. In contrast, the addicted group exhibited consistent negative correlations between sway measures and vertical or rotational dynamics, pointing to possible instability or altered postural strategies, potentially due to changes in sensorimotor integration associated with addiction.

In the 2ec trial (Figure 2B), the non-addicted group demonstrated several moderate correlations between the postural sway metrics and motion variables, indicating specific postural control dynamics under this condition. The CoP_ML displacement showed a moderate positive correlation with vertical displacement (Y), suggesting that increased sway in the ML direction is associated with greater vertical body movement. Similarly, the sway ellipse area was positively correlated with Y, indicating a larger sway area with more vertical movement. Conversely, the ellipse area showed a moderate negative correlation with gyro Z, pointing to a reduction in sway spread with increased vertical axis rotation.

SampEn_ML was moderately positively correlated with gyro Y, suggesting that higher signal complexity in ML sway is associated with greater rotation around the Y-axis. SampEn_AP showed a moderate negative correlation with gyro Z, indicating that simpler or more predictable AP sway is linked to increased vertical rotation. FD in both the ML and AP directions was positively correlated with gyro Y, with a stronger relationship in the AP direction, implying that increased sway complexity is closely tied to rotational motion around the Y-axis.

In the addicted group, a more complex and widespread pattern of significant correlations emerged, involving multiple directions and sensor variables. CoP_ML was positively correlated with gyro Y but negatively with yaw, suggesting an imbalance in ML sway that is influenced differently by rotation around the vertical axis. CoP_AP measures were negatively correlated with X displacement but positively correlated with Z and pitch, indicating that backward–forward sway is reduced with more forward movement but increases with vertical displacement and tilting motion. Total CoP displacement followed similar patterns, showing negative correlation with X but positive correlations with Z, pitch, and gyro Y, reflecting a dynamic postural strategy involving multiple axes.

The ellipse area in the addicted group had strong positive correlations with gyro X and Y, suggesting that broader sway areas coincide with increased frontal and lateral rotations. However, it also showed a negative correlation with gyro Z, indicating less sway with greater vertical axis rotation. SampEn_AP displayed a strong negative correlation with X and strong positive correlations with Z and pitch, reinforcing the notion that AP sway complexity is highly responsive to translational and rotational movement. FD_AP positively correlated with Z and negatively with gyro Z, suggesting sway complexity is influenced by vertical displacement but reduced by vertical rotation.

LyE_AP had strong negative correlations with X and Y but positive correlations with Z and pitch, indicating that greater divergence in AP sway patterns is associated with reduced forward and vertical displacement but increased rotation. In the ML direction, LyE positively correlated with vertical displacement and negatively with gyro X, reflecting the role of both translational and rotational movement in ML sway divergence.

Overall, the non-addicted group showed more focused relationships primarily involving vertical displacement and gyro Y, suggesting controlled and selective integration of sensory input during postural regulation. In contrast, the addicted group displayed a broader and more variable pattern of correlations across multiple planes and sensor inputs, suggesting a more complex or less efficient postural control strategy, potentially indicative of altered sensorimotor integration or compensatory mechanisms related to addiction.

In the eoR trial (Figure 2A), the non-addicted group showed several significant correlations between nonlinear postural measures and motion sensor data. Specifically, there was a moderate positive correlation between FD_ML and gyro Z, indicating that increased complexity in ML sway is associated with greater rotational velocity around the Z-axis. Similarly, LyE_ML correlated moderately with Z displacement, gyro X, and most strongly with gyro Z, suggesting that divergence in ML sway patterns is closely related to both linear and rotational movements. LyE in the AP direction also showed a moderate correlation with gyro Z, further indicating the influence of rotational dynamics on postural control in this group.

In the addicted group, a broader range of significant correlations was observed, and some were stronger than those in the non-addicted group. CoP_ML correlated moderately with gyro X, suggesting a relationship between sway and rotation around the X-axis. SampEn_AP showed a strong correlation with Z displacement and a moderate correlation with gyro X, indicating that signal complexity in AP sway is closely tied to both vertical movement and X-axis rotation. FD in both ML and AP directions showed moderate correlations with gyro Z, aligning with the patterns observed in the non-addicted group. LyE in the ML direction had a relatively strong correlation with gyro X, while LyE in the AP direction correlated moderately with Z displacement.

Overall, the non-addicted group demonstrated a more focused pattern of correlations, particularly involving ML sway and gyro Z, while the addicted group showed more widespread and slightly stronger associations across multiple axes and directions. These results suggest that postural control in addicted individuals may involve altered or compensatory mechanisms that engage a broader range of body movements.

**Figure 3:**
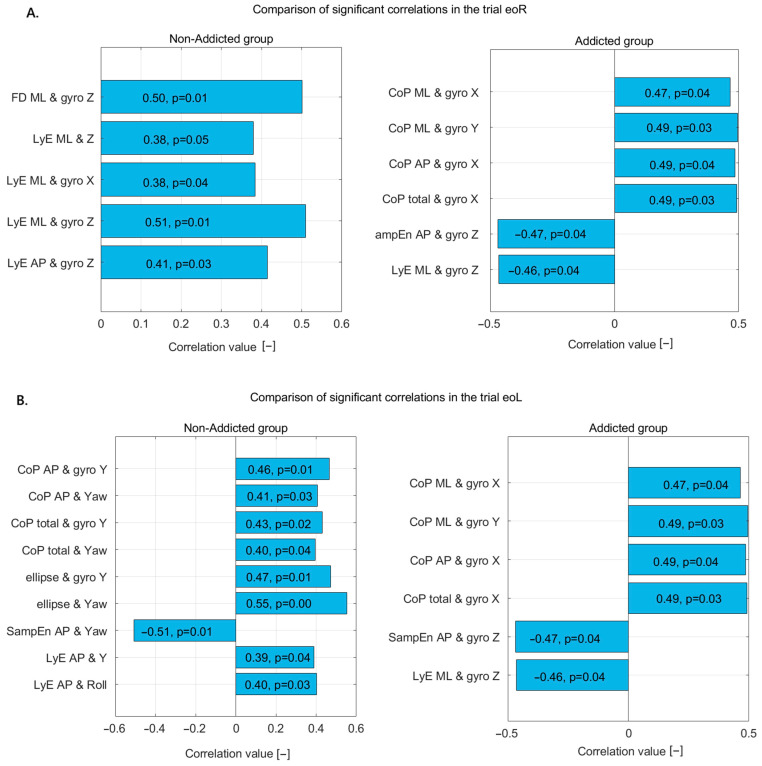
Statistically significant correlations in non-addicted and addicted groups during (**A**) the eoR trial and (**B**) the eoL trial—correlation coefficients and *p*-values displayed on bars.

In the eoL trial (Figure 3B), the non-addicted group displayed several significant correlations between postural variables and sensor-based measures. CoP_AP showed moderate positive correlations with both gyro Y and yaw, suggesting a relationship between AP sway and rotational motion around the vertical axis. Similarly, total CoP displacement was moderately correlated with gyro Y and yaw, further indicating that postural adjustments are linked with rotational dynamics. The ellipse area, which reflects the overall sway range, was also significantly associated with both gyro Y and yaw, with the strongest correlation found with yaw, highlighting the role of horizontal rotational motion in balance maintenance. Interestingly, SampEn_AP had a significant negative correlation with yaw, indicating that greater regularity or less complexity in the AP sway pattern is related to increased rotational activity. Additionally, LyE_AP was moderately correlated with both vertical linear displacement (Y) and roll, suggesting that divergence in AP movement is associated with translational and rotational behaviors, respectively.

In contrast, the addicted group showed a different pattern of significant correlations. CoP in both the ML and AP directions exhibited moderate positive correlations with gyro X and gyro Y, suggesting that sway in these directions is associated with rotational motion around both the frontal and sagittal axes. Total CoP also correlated positively with gyro X, reinforcing the idea that global postural adjustments in individuals with addiction are closely linked to rotational dynamics. Additionally, SampEn_AP showed a significant negative correlation with gyro Z, implying that less-complex AP sway is associated with increased rotation around the vertical axis. LyE_ML direction also showed a negative correlation with gyro Z, suggesting that divergence in ML sway patterns decreases as vertical rotation increases.

Together, these results indicate that non-addicted individuals tend to show stronger associations between postural sway and yaw or vertical displacement, especially in AP measures. In contrast, addicted individuals exhibit broader correlations involving both ML and AP sway with gyro X, Y, and Z. This pattern may reflect different strategies or compensatory mechanisms in postural control between the two groups, possibly influenced by neurophysiological differences related to addiction.

In the ecR trial (Figure 3A), the non-addicted group demonstrated moderate positive correlations between the LyE and body movement variables, suggesting a structured and directionally influenced postural control pattern. Specifically, LyE in the ML direction correlated moderately with vertical displacement (Y) and more strongly with roll, indicating that greater divergence in ML sway is associated with increased vertical movement and lateral trunk rotation. Additionally, LyE in the AP direction showed a moderate positive correlation with roll, implying that greater variability in AP sway also coincides with increased rotational motion around the front-to-back axis.

In contrast, the addicted group exhibited different associations. SampEn in the ML direction showed a moderate positive correlation with gyro Y, suggesting that sway complexity in the ML direction increases with greater rotation around the vertical axis. Similarly, SampEn in the AP direction was moderately correlated with gyro X, indicating that complexity in AP sway increases with rotational motion around the side-to-side axis. Notably, FD in the ML direction showed a strong positive correlation with vertical displacement (Y), suggesting that sway complexity in the ML direction is tightly linked to increased vertical body movement.

These findings indicate that while the non-addicted group primarily relies on rotational dynamics, especially roll, to modulate sway divergence, the addicted group shows stronger relationships between sway complexity and vertical or rotational accelerations, reflecting potentially altered postural control strategies likely linked to the impact of addiction on sensorimotor integration.

**Figure 4:**
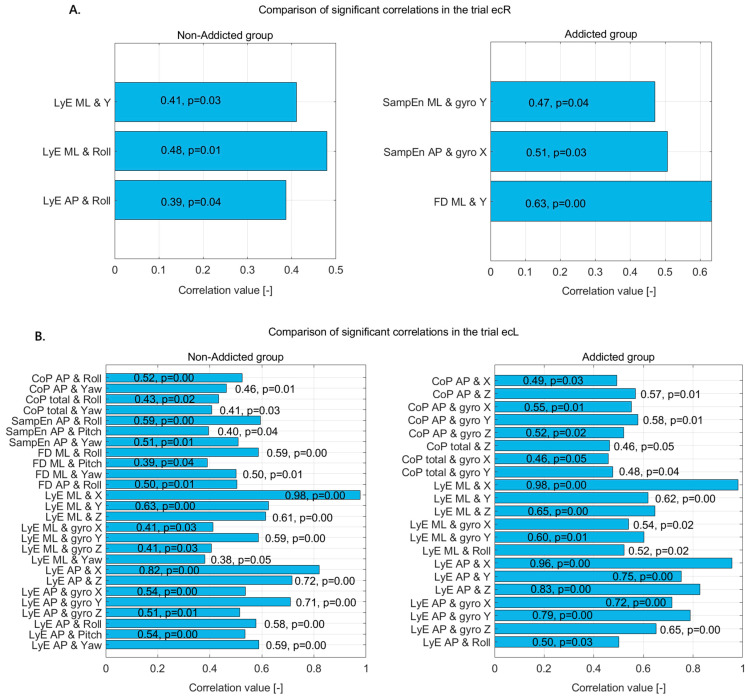
Statistically significant correlations in non-addicted and addicted groups during (**A**) the ecR trial and (**B**) the ecL trial—correlation coefficients and *p*-values displayed on bars.

In the ecL trial (Figure 4B), both the non-addicted and addicted groups demonstrated numerous significant correlations between CoP, sway dynamics, and various kinematic variables, though with distinct patterns and magnitudes.

For the non-addicted group, postural sway measured through CoP in the AP direction showed moderate positive correlations with roll and yaw, indicating that forward–backward sway is influenced by rotational trunk movements. The total CoP movement also correlated with both roll and yaw, reinforcing the influence of rotational dynamics on general postural control.

Complexity and variability measures further highlighted these relationships. SampEn_AP was positively correlated with roll, pitch, and yaw, with the strongest association seen with roll. This suggests that greater postural complexity is linked to rotational movements, particularly around the front–back axis.

FD in the ML and AP directions also showed strong positive correlations with roll, pitch, and yaw, suggesting that sway complexity in both directions is substantially influenced by trunk rotations. Notably, FD_ML had its highest correlation with roll.

The LyE, which captures the divergence and predictability of sway, demonstrated particularly strong associations. In the ML direction, LyE showed a near-perfect correlation with displacement in the X direction and strong correlations with Y, Z, and all three gyroscope axes. Similarly, LyE in the AP direction exhibited strong correlations with X and Z displacements, as well as all gyroscope axes and Euler angles (roll, pitch, and yaw). These results suggest highly coordinated and multi-dimensional control over sway among non-addicted individuals, involving both linear displacements and angular movements.

In the addicted group, a similar pattern of strong correlations emerged, though with some differences in the dominant variables. CoP in the AP direction was positively correlated with displacement in the X and Z axes and with all three gyroscope axes, suggesting that sway in this direction depends significantly on both translational and rotational body movements. CoP total movement also showed significant positive correlations with Z displacement and gyro X and Y, indicating a comparable but slightly less pronounced pattern.

The LyE results in the addicted group closely mirrored those of the non-addicted group. LyE in the ML direction showed a near-perfect correlation with X, and strong correlations with Y, Z, gyro X and Y, and roll. Similarly, LyE in the AP direction displayed very strong correlations with X, Y, Z, and all gyroscope axes, as well as roll. These findings indicate that despite addiction-related changes, the addicted group still relies on comprehensive sensorimotor coordination across multiple planes to control sway.

Overall, while both groups show highly integrated postural control involving translational and rotational components, the non-addicted group appears to have a more distributed pattern across roll, pitch, and yaw, whereas the addicted group shows stronger emphasis on linear and rotational displacements along specific axes. This suggests subtle shifts in motor control strategies, possibly related to altered sensory integration or motor planning in individuals with addiction history.

## 4. Discussion

This study revealed significant differences in postural control between incarcerated individuals with and without a history of substance dependence. Using both linear and nonlinear parameters across varying stance and vision conditions, the key patterns that underscore how addiction-related neurophysiological changes compromise balance and sensorimotor integration are identified. These findings not only align with previous research [8,9,10,11,12] but also offer new insights relevant for correctional rehabilitation.

Importantly, this study focuses on an underexplored and clinically relevant population. Incarcerated individuals, especially those housed in semi-open facilities, represent a group with high rates of substance use disorders, elevated health risks, and limited access to comprehensive medical care. Yet, their functional and neuromuscular deficits, including postural instability, remain largely unaddressed in routine prison healthcare. The semi-open setting offers a more ecologically valid context for assessment, as inmates in these facilities face physical demands more comparable to daily life post-release. This makes postural analysis not only feasible but particularly informative.

Moreover, although this study was conducted in a correctional setting, the findings have broader implications. Similar postural deficits may be expected in individuals undergoing rehabilitation for SUD in outpatient or residential programs. Thus, the assessment protocols used here, particularly those incorporating IMU technology and nonlinear sway analysis, could be adopted in addiction medicine and public health screening initiatives targeting early motor impairments [42].

Our findings also align with the emerging literature showing that incarcerated populations demonstrate decreased physical functioning, higher frailty risk, and altered sensorimotor control even at younger ages [18,19]. These physical impairments, although often overlooked, may contribute to an increased fall risk, reduced participation in rehabilitation, and poorer reintegration outcomes. Functional assessments like the one used in this study may help identify hidden vulnerabilities and guide individualized interventions.

### 4.1. Impairments in Postural Stability Among Addicted Individuals

Consistent with earlier studies, addicted inmates demonstrated significantly poorer postural stability, particularly under conditions that challenge sensory input, such as eyes-closed or single-leg stance trials. Increased sway path length and ellipse area in these tasks suggest that substance-related neurological damage, likely involving cerebellar atrophy and reduced central nervous system integration [43], undermines the body’s ability to maintain balance when visual or proprioceptive cues are limited. The pronounced sway in the AP direction among the addicted group may reflect deficits in anticipatory postural adjustments and dynamic stabilization [2,25].

Furthermore, recent studies employing multiscale sample entropy and other complexity-based metrics have shown that reduced signal complexity in postural sway correlates with impaired sensorimotor integration and diminished neuromuscular adaptability across various motor disorders [32,36]. These nonlinear measures, such as sample entropy and the Lyapunov exponent, serve as sensitive indicators of neuromotor dysfunction and are particularly effective in detecting subtle balance impairments associated with chronic substance abuse. Unlike traditional linear metrics, these complexity-based indices capture the reduced capacity of the neuromotor system to produce flexible and adaptive postural responses.

### 4.2. Impairments in Postural Stability Among Non-Addicted Individuals

While the non-addicted group demonstrated generally better postural control across most conditions, subtle impairments were still observed, particularly under increased sensory or biomechanical challenge. Specifically, trials involving eyes-closed or single-leg stance led to measurable increases in sway path length and ellipse area, indicating that even in individuals without a history of substance abuse, balance can be compromised when visual input is removed or postural demands are heightened.

These fluctuations suggest that although the non-addicted group possessed more-efficient sensorimotor integration compared to their addicted counterparts, their postural control was not immune to destabilizing influences. This may reflect normal physiological limitations in balance regulation, such as the inherent dependency on visual cues or the difficulty of maintaining symmetrical load distribution during asymmetrical stances.

Additionally, nonlinear measures like sample entropy and the Lyapunov exponent showed slight reductions in more demanding conditions, indicating a shift toward more regular and less adaptable sway. While not pathological, these changes highlight the body’s decreased ability to generate flexible postural responses when sensory feedback is restricted.

The IMU-based analysis revealed relatively stable trunk movement patterns; however, modest increases in mediolateral angular velocity during single-leg and eyes-closed tasks suggest that even non-addicted individuals may rely on compensatory strategies involving trunk rotation and repositioning. These adaptations, while effective in maintaining upright posture, indicate that postural control under challenge is an active and dynamic process, even in neurologically intact populations.

Taken together, these findings underscore the importance of task complexity in revealing latent instability. They also emphasize the need for balance assessments that include multiple conditions, as a static two-legged stance with eyes open, which may overlook subtle postural control limitations, even in healthy individuals.

### 4.3. Linear vs. Nonlinear Analysis: Complementary Perspectives

While traditional linear metrics such as CoP path length effectively captured the magnitude of sway, nonlinear measures (e.g., sample entropy, fractal dimension, and the Lyapunov exponent) provided deeper insights into the adaptability, structure, and temporal complexity of postural control. Addicted individuals showed lower SampEn and LyE values in several trials, especially during the 2ec (bipedal eyes-closed) condition, indicating more rigid and less adaptable control strategies. This could reflect impaired neuromotor coordination and disrupted sensorimotor feedback loops, a pattern common in aging or neurologically compromised populations [2,25].

Interestingly, FD values trended higher among addicted participants, suggesting a shift toward more chaotic or disorganized sway. Although these differences were not statistically significant, they point to potential alterations in central balance regulation, echoing findings from other clinical populations such as obese children [44].

### 4.4. Kinematic Correlations Reveal Diverging Postural Strategies

This section examines the relationship between postural sway measures and inertial sensor-derived kinematic data in individuals with and without a history of addiction, highlighting key differences in motor strategies, sensorimotor integration, and postural adaptability. In the non-addicted group, during the 2eo trial (eyes open and symmetrical stance), sway metrics such as sample entropy (SampEn) and fractal dimension (FD) in mediolateral (ML) and anteroposterior (AP) directions correlated strongly with precise trunk rotations (pitch and roll) and linear displacements (X and Y). For example, SampEn_ML positively correlated with gyro X, indicating that increased sway complexity relates to higher sagittal plane trunk rotation, reflecting fine-tuned trunk adjustments for balance. Meanwhile, a weaker negative correlation with gyro Z suggested that minimizing axial rotation stabilizes sway. In the AP direction, SampEn negatively correlated with linear displacement and positively with pitch, showing that sway becomes more predictable when trunk tilts rather than large horizontal shifts stabilize posture. Similar patterns were seen with fractal dimension and the Lyapunov exponent, indicating a coordinated, biomechanically efficient postural control strategy relying on well-integrated sensorimotor feedback loops.

In contrast, the addicted group displayed more variable, widespread correlations across multiple axes, lacking biomechanical specificity. For instance, CoP_ML negatively correlated with gyro Y, suggesting reduced lateral trunk rotation is linked to greater ML sway, possibly due to insufficient compensation. The sway ellipse area negatively correlated with vertical displacement (Z), roll, and gyro Y, implying a rigid, inefficient strategy where certain movements are restricted, leading to broader but uncontrolled sway. Negative correlations between FD_ML and vertical displacement further indicate poor integration between linear and angular control. These diffuse and paradoxical patterns likely reflect compensatory rigidity, impaired sensory integration, or neuromuscular inefficiency, consistent with addiction-related neurological impairments [11,12].

Under sensory-challenging conditions (2ec trial and eyes closed), these differences became more pronounced. The non-addicted group maintained adaptive control, with CoP_ML and ellipse area positively correlating with vertical displacement, suggesting active modulation of sway via trunk and knee flexion, and negative correlations with gyro Z indicating strategic axial rotation to limit sway. Nonlinear measures remained linked with gyro Y, highlighting the use of rotational degrees of freedom under reduced sensory input. Conversely, the addicted group exhibited disorganized, inconsistent correlations. CoP_ML showed conflicting relationships with gyro Y and yaw, while CoP_AP correlated with linear and rotational variables, suggesting overcompensation or instability. The ellipse area’s positive correlation with gyro X and Y, alongside negative correlation with gyro Z, pointed to excessive, uncontrolled trunk motion and limited axial stabilization. Nonlinear measures further supported this disrupted postural regulation, with complex and inconsistent correlations across multiple kinematic dimensions.

Overall, non-addicted individuals demonstrated coordinated and efficient postural strategies marked by coherent sway-kinematic relationships, reflecting preserved sensorimotor integration and balance control. In contrast, addicted individuals showed dispersed, maladaptive control patterns characterized by overreliance on compensatory trunk movements and disrupted coordination. These findings underscore the importance of analyzing not just sway magnitude but also the functional coordination between sway patterns and kinematic control, especially in neurologically vulnerable populations [11,12,24].

### 4.5. Comparative Summary of Findings

The findings are summarized in Table 6, which contrasts the postural control characteristics observed in the non-addicted and addicted groups. Across all domains, linear parameters, nonlinear dynamics, and kinematic correlations, addiction was associated with degraded postural performance, particularly under conditions of increased sensory or biomechanical challenge.

### 4.6. Clinical and Rehabilitation Implications

These results have important implications for prison healthcare systems. While most correctional programs prioritize psychological and behavioral therapies, our findings emphasize the need to also address physical sequelae of substance abuse, particularly motor control deficits that may increase fall risk, reduce functional independence, and hinder reintegration outcomes.

The use of multi-modal and portable technologies, such as the STANIAK JB platform and BTS G-Sensor IMU, provides an efficient and objective method for assessing balance. This dual-system approach not only ensures cross-validation but also facilitates real-time monitoring, making it a viable tool for routine screenings in semi-open prison settings.

These findings support the broader application of postural assessments in addiction medicine. Similar impairments may be observed in individuals undergoing treatment in outpatient or inpatient rehabilitation centers, where early detection of balance deficits could inform physiotherapeutic or neurorehabilitation strategies. Moreover, incorporating such assessments into public health programs could help identify at-risk individuals with a history of substance use before major functional decline occurs [45,46].

### 4.7. Sociodemographic Considerations

Though demographic differences between groups were not statistically significant, trends toward lower education and higher divorce rates among addicted individuals mirror known links between social determinants and addiction. These findings highlight the broader psychosocial vulnerabilities in this population, reinforcing the importance of holistic, interdisciplinary rehabilitation strategies.

### 4.8. Study Limitation

Several limitations should be acknowledged in this study. The relatively small sample size and confinement to a single correctional facility limit the generalizability of the results. In addition, the cross-sectional design prevents causal conclusions from being drawn regarding the impact of substance use on postural control. Another significant limitation is the absence of a standardized and universally accepted measure of addiction severity, which may have introduced variability in how participants’ substance use histories were classified and interpreted. Additionally, only a single trial was conducted for each of the six balance conditions, whereas several papers [47,48] recommend performing at least three trials per condition to ensure measurement reliability. This represents a potential source of bias, the impact on the results of which cannot currently be quantified. It is suggested that future longitudinal research be conducted to track the progression of balance recovery during and after addiction treatment.

It should also be noted that the prison environment imposed significant restrictions. Access to information was limited due to legal requirements (e.g., court rulings), privacy protections (e.g., GDPR), and institutional policies. Permission to conduct this study within a correctional facility was obtained only after a challenging and complex approval process, and the successful completion of data collection under these conditions is considered a major accomplishment. The sample size was further limited by logistical and administrative constraints, such as the need for continuous supervision by correctional staff and individual authorizations from prison authorities. As a result, the recruitment of a larger or more randomized sample was not possible.

Due to these limitations, a formal a priori sample size calculation was not carried out. To reflect the exploratory nature of this research, the title of this manuscript has been revised to include the term “*pilot study*”.

Abstinence at the time of testing was confirmed by prison healthcare personnel for all participants. However, the duration of abstinence varied among individuals, and although partial data were available, it was not consistently recorded. This has been recognized as a limitation in the revised manuscript.

Finally, it is recommended that future research investigate the potential benefits of physical rehabilitation strategies, such as proprioceptive, vestibular, or compensatory balance training, that have demonstrated effectiveness in athletic populations. Their applicability within correctional settings should be explored as a means to reduce postural control deficits among individuals with a history of substance dependence.

## 5. Conclusions

### 5.1. Main Findings

This study provides compelling evidence that substance dependence is associated with significant impairments in postural control, particularly under conditions that challenge sensory integration and neuromuscular coordination. By employing both linear and nonlinear stabilometric parameters alongside inertial measurement unit (IMU) kinematic data, the research uncovered clear differences in balance regulation strategies between incarcerated individuals with and without a history of addiction. The addicted group consistently demonstrated greater postural sway; reduced stability; and less-efficient, multi-axis control patterns—indicative of compromised sensorimotor function likely stemming from long-term neurophysiological changes. These findings confirm that substance dependence negatively impacts postural control, with measurable deficits observable through both linear and nonlinear balance metrics.

### 5.2. Clinical Considerations

These findings highlight the critical need to broaden rehabilitation strategies within correctional facilities to include comprehensive physical assessments and targeted motor interventions. Traditional approaches focusing solely on psychological or behavioral recovery may overlook persistent functional deficits, such as impaired postural control, which can limit daily functioning and independence, potentially complicating reintegration into society and indirectly increasing the risk of recidivism. The integration of advanced posturographic tools and kinematic analyses into prison healthcare protocols may facilitate early identification of motor impairments and support the development of individualized rehabilitation plans.

Incorporating physical rehabilitation, particularly exercises aimed at improving proprioceptive, vestibular, and neuromuscular function, could play a pivotal role in restoring functional independence and enhancing overall rehabilitation outcomes. Future research should prioritize longitudinal studies to track recovery trajectories and assess the effectiveness of targeted interventions designed to improve postural control in incarcerated individuals with a history of substance dependence. Interventions such as structured physical activity programs, balance training, and neurofeedback have shown promise in other confined or neurologically vulnerable populations. For example, physical rehabilitation significantly improved both psychological and physiological outcomes among World War II prisoners of war [49]. Additionally, emerging approaches like EEG biofeedback may provide innovative methods to enhance motor performance by modulating central nervous system activity and promoting neural adaptability [50]. Neuroimaging techniques could further enhance our understanding of the structural and functional changes associated with postural instability in individuals with substance addiction, particularly within cerebellar and fronto-striatal circuits that are involved in motor coordination and executive control [10,12].

Importantly, future studies should explore the applications of interpretable machine learning and generative modeling to postural or gait analysis using wearable sensors [51,52,53,54]. Recent research demonstrates that these approaches can improve both classification performance and clinical insights by incorporating variability- and complexity-based features such as approximate entropy (ApEn), the Lyapunov exponent, and sample entropy. Integrating this emerging literature would further strengthen the scientific framework and translational potential of postural control research in addiction.

Ultimately, addressing the physical consequences of addiction is crucial for developing a more comprehensive and integrative model of correctional healthcare.

## Figures and Tables

**Table 1:** Characteristics of participants reported as median (Q1—first quartile; Q3—third quartile).

Group	Age [Years]	Body Mass [kg]	Body Height [cm]	Time Spent in Prison [Months]	Time Spent in Semi-Open Prison [Months]
Addicted (N = 19)	21.6 (19.6; 24.3)	86.7 (78.7; 94.4)	177 (173; 183)	10 (5.5; 19)	7 (1; 10)
Non-Addicted (N = 28)	26.5 (21.1; 30)	81.5 (74.3; 91)	178.5 (176; 185)	8 (3.25; 17.25)	3.5 (1.5; 6.5)
Male (N = 47)	24.3 (20.1; 29.4)	82.9 (74.5; 93.3)	178 (175; 184)	9 (3.5; 18)	4 (1.5; 9)

**Table 2:** Addiction characteristics and treatment outcomes among incarcerated individuals.

Addiction Type	Duration of Use	Therapy Status	Facility	Engagement Level	Notes
Alcohol	~11 years	Currently in therapy	OT Wojkowice	ND	
	~14 years	Completed	OT Wojkowice	High	
	~16 years	Completed	OT Wojkowice	High	
	Several years	Completed	OT Wojkowice	High	
	~10 years	Completed	OT Wojkowice	High	
	~20 years (2005–2025)	Completed	OT Wojkowice	Moderate	
	~4 years	Completed	OT Jasło	Moderate	
	~10 years + relapse	Completed	ND	Moderate	Abstinent for 10 years before relapse
	~29 years	Abstinent (6 years)	ND	ND	No therapy during incarceration
	~11 years	Completed	ND	Minimal	Limited behavioral change observed
	~3 years	Outpatient therapy (2020)	ND	ND	Participated in reintegration program
	~10 years	Completed	OT Wojkowice	ND	
Psychoactive substances	~5 years	Completed	Unspecified	Moderate	
	~2 years	Awaiting therapy (Nov 2025)	OT Wojkowice		Court-mandated
	~3 years (estimated)	Abstinent	ND		No therapy during incarceration
α-PVP	~2 years	Completed	OT Suwałki	High	
Amphetamine	~5 years	Completed	OT Przemyśl	High	Post-relapse treatment
Cannabinoids	~5 years	Abstinent (1.5 years)			Participated in preventive program
Alcohol + Drug	Various	Completed	OT Wojkowice	High	Co-occurring addiction

OT—Therapeutic Unit; ND—no data.

**Table 3:** Median (Q1—first quartile; Q3—third quartile), Z—value, and effect size (r) for the linear postural sway parameters in addicted and non-addicted groups across various balance conditions, where * indicates statistically significant differences.

Trial	Variable	Non-Addicted Group Median (Q1; Q3)	Addicted Group Median (Q1; Q3)	Z	*p*-Value	Effect Size (R)
2eo	CoP_total [mm]	257 (222.5; 297.5)	240 (223; 317)	0.13	0.89	0.02
	CoP_ML [mm]	130.5 (112.5; 148.5)	108 (91; 147)	1.36	0.19	0.2
	CoP_AP [mm]	190 (162; 230.5)	187 (155; 270)	−0.36	0.71	−0.05
	ellipse [mm^2^]	476 (345.5; 672.5)	411 (233; 526)	1.32	0.18	0.19
2ec	CoP_total [mm]	364 (305; 434)	459 (364; 601)	−2.40	0.01 *	−0.35
	CoP_ML [mm]	138 (117; 168)	161 (129; 202)	−1.58	0.11	−0.23
	CoP_AP [mm]	314.5 (248.5; 372)	399 (301; 494)	−2.46	0.01 *	−0.36
	ellipse [mm^2^]	538 (368; 894.5)	814 (619; 975)	−2.11	0.03 *	−0.31
eoR	CoP_total [mm]	1159 (918.5; 1280)	1479 (1061; 2374)	−2.08	0.03 *	−0.3
	CoP_ML [mm]	718 (586.5; 900)	925 (622; 1474)	−2.12	0.03 *	−0.31
	CoP_AP [mm]	714.5 (630.5; 816)	828 (668; 1489)	−2.35	0.01 *	−0.34
	ellipse [mm^2^]	4997.5 (3784.5; 7325.5)	9239 (4734; 14,427)	−1.83	0.06	−0.27
eoL	CoP_total [mm]	1009 (803; 1186.5)	1141 (983; 1702)	−2.35	0.01 *	−0.34
	CoP_ML [mm]	605 (531; 748)	774 (671; 1249)	−2.69	0.01 *	−0.39
	CoP_AP [mm]	632.5 (519.5; 777.5)	763 (670; 979)	−2.07	0.03 *	−0.3
	ellipse [mm^2^]	4054.5 (2925.5; 5453)	5312 (3291; 7242)	−1.33	0.18	−0.19
ecR	CoP_total [mm]	3064.5 (2335; 3701)	4579 (3376; 5604)	−2.61	0.01 *	−0.38
	CoP_ML [mm]	2090 (1631; 2385.5)	3089 (2058; 3774)	−2.33	0.01 *	−0.34
	CoP_AP [mm]	1829 (1376.5; 2388.5)	2463 (2135; 3808)	−2.66	0.01 *	−0.39
	ellipse [mm^2^]	28,023.5 (16,486.5; 48,033)	46,599 (34,925; 138,897)	−2.56	0.01 *	−0.37
ecL	CoP_total [mm]	3085.5 (2549; 3805)	4163 (3256; 5194)	−2.63	0.01 *	−0.38
	CoP_ML [mm]	2013.5 (1782; 2533.5)	2516 (2180; 3621)	−2.31	0.02 *	−0.34
	CoP_AP [mm]	1822.5 (1424.5; 2386)	2374 (1949; 2996)	−2.54	0.01 *	−0.37
	ellipse [mm^2^]	31,509.5 (22,541.5; 57,632)	52,054 (28,035; 84,206)	−1.70	0.08	−0.25

CoP—center of pressure, ML—mediolateral direction, AP—anterior–posterior direction, 2eo—bipedal standing with eyes open, 2ec—bipedal standing with eyes closed, eoR—right-leg standing with eyes open, eoL—left-leg standing with eyes open, ecR—right-leg standing with eyes closed, ecL—left-leg standing with eyes closed.

**Table 4:** Median (Q1—first quartile; Q3—third quartile), Z—value, and effect size (r) for the nonlinear postural sway parameters in addicted and non-addicted groups across various balance conditions, where * indicates statistically significant differences.

Trial	Variable	Non-Addicted Group Median (Q1; Q3)	Addicted Group Median (Q1; Q3)	Z	*p*-Value	Effect Size (r)
2eo	SampEn_ML [-]	0.08 (0.06; 0.11)	0.09 (0.06; 0.11)	−0.07	0.93	−0.01
	SampEn_AP [-]	0.05 (0.04; 0.06)	0.07 (0.05; 0.1)	−1.7	0.07	−0.25
	FD_ML [-]	1.32 (1.24; 1.39)	1.29 (1.27; 1.36)	0.53	0.59	0.08
	FD_AP [-]	1.24 (1.2; 1.27)	1.29 (1.23; 1.33)	−1.87	0.06	−0.27
	LyE_ML [-]	1.04 (0.89; 1.2)	1.05 (0.81; 1.16)	0.68	0.49	0.1
	LyE_AP [-]	1.34 (1.29; 1.46)	1.37 (1.28; 1.52)	−0.42	0.67	−0.06
2ec	SampEn_ML [-]	0.12 (0.08; 0.15)	0.12 (0.07; 0.16)	−0.05	0.95	−0.01
	SampEn_AP [-]	0.08 (0.06; 0.09)	0.1 (0.08; 0.12)	−2.04	0.04 *	−0.3
	FD_ML [-]	1.35 (1.27; 1.41)	1.34 (1.27; 1.36)	0.70	0.48	0.1
	FD_AP [-]	1.27 (1.24; 1.31)	1.32 (1.25; 1.39)	−1.94	0.05	−0.28
	LyE_ML [-]	0.97 (0.88; 1.11)	1.16 (0.89; 1.25)	−1.48	0.13	−0.22
	LyE_AP [-]	1.52 (1.41; 1.64)	1.64 (1.5; 1.74)	−2.41	0.01 *	−0.35
eoR	SampEn_ML [-]	0.16 (0.11; 0.22)	0.14 (0.12; 0.24)	−0.24	0.80	−0.04
	SampEn_AP [-]	0.12 (0.09; 0.15)	0.13 (0.11; 0.18)	−0.94	0.34	−0.14
	FD_ML [-]	1.44 (1.37; 1.49)	1.44 (1.39; 1.49)	−0.20	0.83	−0.03
	FD_AP [-]	1.41 (1.37; 1.47)	1.45 (1.39; 1.48)	−0.98	0.32	−0.14
	LyE_ML [-]	1.91 (1.74; 1.95)	1.9 (1.78; 2.04)	−0.85	0.39	−0.12
	LyE_AP [-]	1.88 (1.83; 1.97)	1.92 (1.79; 2.03)	−0.44	0.65	−0.06
eoL	SampEn_ML [-]	0.15 (0.12; 0.18)	0.19 (0.13; 0.24)	−1.72	0.08	−0.25
	SampEn_AP [-]	0.11 (0.09; 0.14)	0.12 (0.1; 0.16)	−0.83	0.40	−0.12
	FD_ML [-]	1.46 (1.38; 1.49)	1.47 (1.4; 1.52)	−0.83	0.40	−0.12
	FD_AP [-]	1.42 (1.37; 1.46)	1.43 (1.4; 1.47)	−1.33	0.18	−0.19
	LyE_ML [-]	1.87 (1.79; 2.01)	1.88 (1.8; 1.99)	0.05	0.95	0.01
	LyE_AP [-]	1.86 (1.73; 1.96)	1.94 (1.74; 2.03)	−0.79	0.42	−0.12
ecR	SampEn_ML [-]	0.12 (0.09; 0.16)	0.13 (0.07; 0.15)	0.89	0.36	0.13
	SampEn_AP [-]	0.14 (0.12; 0.17)	0.15 (0.12; 0.18)	0.11	0.90	0.02
	FD_ML [-]	1.38 (1.35; 1.43)	1.36 (1.33; 1.4)	1.48	0.13	0.22
	FD_AP [-]	1.41 (1.35; 1.45)	1.4 (1.36; 1.45)	−0.68	0.49	−0.1
	LyE_ML [-]	2.11 (2.04; 2.2)	2.08 (2.06; 2.2)	0.40	0.68	0.06
	LyE_AP [-]	2.12 (2.03; 2.21)	2.14 (2.07; 2.24)	−1.26	0.20	−0.18
ecL	SampEn_ML [-]	0.13 (0.1; 0.16)	0.12 (0.08; 0.16)	0.44	0.65	0.06
	SampEn_AP [-]	0.14 (0.12; 0.16)	0.15 (0.12; 0.17)	−0.22	0.81	−0.03
	FD_ML [-]	1.39 (1.37; 1.42)	1.37 (1.32; 1.41)	1.48	0.13	0.22
	FD_AP [-]	1.42 (1.38; 1.48)	1.4 (1.35; 1.43)	1.68	0.09	0.25
	LyE_ML [-]	2.16 (2.09; 2.23)	2.15 (2.07; 2.23)	0.59	0.55	0.09
	LyE_AP [-]	2.17 (2.07; 2.2)	2.15 (2.06; 2.22)	0.20	0.83	0.03

ML—mediolateral direction, AP—anterior–posterior direction, SampEn—sample entropy, FD—fractal dimension, LyE—Lyapunov exponent, 2eo—bipedal standing with eyes open, 2ec—bipedal standing with eyes closed, eoR—right-leg standing with eyes open, eoL—left-leg standing with eyes open, ecR—right-leg standing with eyes closed, ecL—left-leg standing with eyes closed.

**Table 5:** Median (Q1—first quartile; Q3—third quartile) for the median values of waveforms from IMU data in addicted and non-addicted groups across various balance conditions, where * indicates statistically significant differences, Z—value, and effect size (r).

Trial	Variable	Non-Addicted Group Median (Q1; Q3)	Addicted Group Median (Q1; Q3)	Z	*p*-Value	Effect Size (r)
2eo	X [deg/s^2^]	9.7 (9.64; 9.76)	9.65 (7.57; 9.71)	2.00	0.04 *	0.29
	Y [deg/s^2^]	−0.02 (−0.32; 0.26)	−0.38 (−0.59; −0.03)	2.42	0.01 *	0.35
	Z [deg/s^2^]	−0.24 (−0.87; 0.77)	1.2 (0.24; 1.71)	−2.49	0.01 *	−0.36
	gyro X [deg/s]	0 (0; 0)	0 (0; 0)	0.81	0.41	0.12
	gyro Y [deg/s]	0 (0; 0)	0 (−0.01; 0)	0.29	0.76	0.04
	gyro Z [deg/s]	0 (0; 0)	0 (0; 0)	−0.40	0.68	−0.06
	Roll [deg]	2.6 (−84.1; 57.18)	−34.2 (−65.35; −6.3)	1.76	0.07	0.26
	Pitch [deg]	−82.28 (−85.05; −79.13)	−79.3 (−82.85; −64.37)	−2.30	0.02 *	−0.34
	Yaw [deg]	−37.73 (−98.93; 30)	15.12 (−40.4; 69.7)	−1.70	0.08	−0.25
2ec	X [deg/s^2^]	9.69 (9.61; 9.78)	9.66 (9.62; 9.73)	1.10	0.26	0.16
	Y [deg/s^2^]	−0.01 (−0.58; 0.67)	−0.22 (−0.48; 0.2)	0.85	0.39	0.12
	Z [deg/s^2^]	0.23 (−1.49; 1.26)	0.3 (−0.15; 1.61)	−1.09	0.27	−0.16
	gyro X [deg/s]	0 (0; 0)	0 (0; 0)	0.10	0.91	0.01
	gyro Y [deg/s]	0 (−0.06; 0)	−0.03 (−0.03; 0)	−0.33	0.74	−0.05
	gyro Z [deg/s]	0 (0; 0)	0 (0; 0)	−0.29	0.77	−0.04
	Roll [deg]	−0.3 (−128.6; 49.8)	−34.68 (−78; 3.8)	0.48	0.62	0.07
	Pitch [deg]	−82.6 (−84.6; −77.5)	−80.48 (−83.04; −78.6)	−1.06	0.28	−0.15
	Yaw [deg]	−35.7 (−127.3; 119.4)	21.61 (−54.6; 58.6)	−0.43	0.66	−0.06
eoR	X [deg/s^2^]	9.71 (9.63; 9.77)	9.66 (9.61; 9.73)	1.33	0.18	0.19
	Y [deg/s^2^]	0.01 (−0.4; 0.61)	0.08 (−0.15; 0.4)	−0.30	0.76	−0.04
	Z [deg/s^2^]	0.23 (−1.44; 1.06)	0.36 (−0.43; 1.67)	−1.28	0.20	−0.19
	gyro X [deg/s]	0 (0; 0)	0.03 (0; 0.09)	−1.95	0.04 *	−0.28
	gyro Y [deg/s]	0 (−0.06; 0.06)	−0.03 (−0.08; 0.06)	0.25	0.80	0.04
	gyro Z [deg/s]	0 (−0.06; 0.06)	0 (0; 0.03)	−0.54	0.58	−0.08
	Roll [deg]	13 (−56.4; 154.9)	25.11 (−5.45; 50.95)	−0.09	0.92	−0.01
	Pitch [deg]	−81.2 (−85.8; −79.1)	−80.14 (−82.8; −78.15)	−1.24	0.21	−0.18
	Yaw [deg]	17.2 (−122.85; 62.7)	−23.05 (−59.3; 40.05)	−0.06	0.94	−0.01
eoL	X [deg/s^2^]	9.69 (9.62; 9.74)	9.66 (9.57; 9.71)	0.80	0.42	0.12
	Y [deg/s^2^]	0.47 (−1.18; 1.07)	−0.52 (−0.96; −0.03)	1.36	0.17	0.2
	Z [deg/s^2^]	0.04 (−1.25; 1.02)	0.4 (−0.19; 1.44)	−1.17	0.23	−0.17
	gyro X [deg/s]	0 (0; 0)	0 (0; 0.06)	0.29	0.77	0.04
	gyro Y [deg/s]	0 (−0.12; 0.12)	0 (−0.06; 0.06)	0.29	0.77	0.04
	gyro Z [deg/s]	0 (0; 0)	0 (0; 0.03)	−0.33	0.74	−0.05
	Roll [deg]	25.3 (−128.4; 49.4)	−35.51 (−56.8; −5.48)	0.54	0.58	0.08
	Pitch [deg]	−80.8 (−82.8; −80)	−80.26 (−81.6; −77.1)	−0.64	0.51	−0.09
	Yaw [deg]	−43.5 (−109.5; 89.9)	−1.9 (−51.8; 58.68)	−0.30	0.76	−0.04
ecR	X [deg/s^2^]	9.65 (9.55; 9.76)	9.65 (9.59; 9.71)	0.51	0.60	0.07
	Y [deg/s^2^]	−0.02 (−0.42; 0.38)	−0.08 (−0.77; 0.45)	0.39	0.69	0.06
	Z [deg/s^2^]	0.32 (−1.13; 1.41)	0.33 (−0.37; 1.25)	−0.77	0.43	−0.11
	gyro X [deg/s]	−0.06 (−0.06; 0.06)	0 (−0.05; 0.12)	−1.19	0.23	−0.17
	gyro Y [deg/s]	0 (−0.06; 0.12)	0 (−0.06; 0.09)	0.30	0.75	0.04
	gyro Z [deg/s]	0 (−0.12; 0)	0 (−0.09; 0.03)	−0.77	0.43	−0.11
	Roll [deg]	−1.8 (−82.9; 80.2)	−18.77 (−51.45; 34.2)	0.22	0.82	0.03
	Pitch [deg]	−79.6 (−83.3; −77.1)	−80.5 (−82.15; −77.03)	−0.35	0.72	−0.05
	Yaw [deg]	−57.5 (−114.8; 34.05)	−47.83 (−56.7; −2.29)	−0.57	0.56	−0.08
ecL	X [deg/s^2^]	9.69 (9.55; 9.71)	9.68 (9.56; 9.71)	0.51	0.60	0.07
	Y [deg/s^2^]	−0.03 (−1.41; 0.35)	−0.64 (−0.96; −0.33)	0.54	0.58	0.08
	Z [deg/s^2^]	−1.12 (−1.88; −0.06)	0.02 (−0.97; 0.41)	−1.99	0.04 *	−0.29
	gyro X [deg/s]	0 (−0.12; 0.06)	−0.05 (−0.12; 0.03)	0.71	0.47	0.1
	gyro Y [deg/s]	0.06 (0; 0.18)	0.03 (−0.06; 0.15)	0.52	0.59	0.08
	gyro Z [deg/s]	0 (−0.06; 0.06)	0 (−0.06; 0.03)	−0.11	0.90	−0.02
	Roll [deg]	−74.5 (−140.2; 152.5)	−33.63 (−67.88; 12.6)	−0.33	0.74	−0.05
	Pitch [deg]	−79.6 (−81.3; −75.7)	−80.4 (−82.4; −76.85)	0.31	0.75	0.05
	Yaw [deg]	15.5 (−70.7; 54.4)	3.48 (−41.1; 68.95)	−0.05	0.95	−0.01

2eo—bipedal standing with eyes open, 2ec—bipedal standing with eyes closed, eoR—right-leg standing with eyes open, eoL—left-leg standing with eyes open, ecR—right-leg standing with eyes closed, ecL—left-leg standing with eyes closed. Angular acceleration was recorded along three axes: X [deg/s^2^], where a positive value indicates acceleration to the right; Y [deg/s^2^], where a positive value reflects acceleration forward; and Z [deg/s^2^], where a positive value represents acceleration upward. Angular velocity was measured as gyro X [deg/s], gyro Y [deg/s], and gyro Z [deg/s], corresponding to rotation rates around the X (mediolateral), Y (anterior–posterior), and Z (perpendicular/vertical) axes, respectively. Orientation angles included roll [deg], indicating rotation around the X-axis (rightward tilt is positive); pitch [deg], indicating rotation around the Y-axis (forward tilt is positive); and yaw [deg], indicating rotation around the Z-axis (clockwise turn is positive). These directions and sign conventions match those used for angular acceleration.

**Table 6:** Comparative summary of postural control findings.

Feature	Non-Addicted Group	Addicted Group
Linear parameters	Generally stable, minimal sway in simple tasks.	Increased sway (CoP_total, CoP_AP, ellipse) in challenging tasks (eyes closed and single-leg).
Nonlinear measures	Predictable and selective sway complexity (high SampEn and FD) associated with specific motion dynamics.	Higher SampEn and LyE in the AP direction during eyes-closed bipedal stance, indicating more irregular and unstable control.
IMU kinematics	More focused and consistent correlation patterns; stable rotational modulation of sway divergence.	Greater vertical and mediolateral movement (pitch angle and angular velocity) even in simple tasks, suggesting altered postural alignment and motor noise.
Interpretation	Stable sensorimotor integration, and efficient and structured postural regulation.	Impaired sensory integration, reduced neuromuscular control, and altered postural dynamics, especially when visual input is absent or postural demands are high.

## Data Availability

The data presented in this study are available from the corresponding author on request. The data are not publicly available due to ongoing data collection and further research being conducted on this topic.
